# Supplementary material for: Escherichia coli possessing the dihydroxyacetone phosphate shunt utilize 5′-deoxynucleosides for growth
Source: Microbiol Spectr. 2024 Mar 5;12(4):e03086-23. doi: 10.1128/spectrum.03086-23 (PMC10986504; doi:10.1128/spectrum.03086-23)
Supplement: Supplemental material — Tables S1 and S2 and Figures S1 to S6. [file spectrum.03086-23-s0001.docx]

**Supplementary Information**

**Supplementary Tables:**

**Supplementary Table S1.** Primers and Plasmids Used in this Study

| **Fragment** | **Description** | **Primer name** | **Sequence (5’-3’): *E. coli* sequence underlined** |
| --- | --- | --- | --- |
| λRed-MtnN | PCR fragment for deletion of *pfs* gene by λ-Red recombinase | 25922-MtnN-F | GAGTAAATCTATGAAAATCGGCATCATTGGTGCAATGGAA GTGTAGGCTGGAGCTGCTTC |
|  |  | 25022-MtnN-R | GAACAGTGACTTAGCCATGTGCCAGTTTCTGCACCAGTGA CATATGAATATCCTCCTTAGTTCC |
| λRed-K2 | PCR fragment for deletion of *mtnK*, *mtnA*, and *ald2* by λ-Red recombinase | 25922-MtnK-F | GAGAACTGTTATGACGGATTCAATTCCATCAGGTTACAAG GTGTAGGCTGGAGCTGCTTC |
|  |  | 25922-Ald2-R | AAAGCTGTAATTACGCTTTCAAACCATACGTTTTGAATTT CATATGAATATCCTCCTTAGTTCCTATTCC |
|  |  |  |  |
| **Plasmid** | **Description** | **Primer name** | **Sequence (5’-3’): restriction site underlined** |
| pTETTET | Expression plasmid with the tetracycline-inducible promoter Pzt-1 | Pzt1RBS-AseI-F | gcagattaatgccccatacgatataagttgtaattc |
|  |  | Pzt1RBS-NdeI-R | gcagcatatgcgactttctcctctttaatgaattc |
| pK2 | pTETTET expression plasmid for DHAP shunt kinase (MtnK; *mtnK* gene product), isomerase (MtnA; *mtnA* gene product), and aldolase (Ald2; *ald2* gene product) | K2-NdeI-F | GCAGTCCATATGACGGATTCAATTCCATCAGG |
|  |  | K2-SacI-R | GCTGACGAGCTCTTACGCTTTCAAACCATACGTTTTG |
| pPfs | pTETTET expression plasmid for *E. coli* Pfs nucleosidase | Pfs-NdeI-F | CATCGTCATATGATGAAAATCGGCATCATTGGTGCAATG |
|  |  | Pfs-SacI-R | CATCGTGAGCTCTTAGCCATGTGCCAGTTTCTGCAC |

**Supplementary Table S2:** *Synthesized Tet Promoter Sequence (with AseI and NdeI sites underlined)* *for complementation studies:*

| **Tetracycline (tet) Promoter Sequence** |
| --- |
| attaatTCTAAAGGGTGGTTAACTCGACATCTTGGTTACCGTGAAGTTACCATCACGGAAAAAGGTTATGCTGCTTTTAAGACCCACTTTCACATTTAAGTTGTTTTTCTAATCCGCAGATGATCAATTCAAGGCCGAATAAGAAGGCTGGCTCTGCACCTTGGTGATCAAATAATTCGATAGCTTGTCGTAATAATGGCGGCATACTATCAGTAGTAGGTGTTTCCCTTTCTTCTTTAGCGACTTGATGCTCTTGATCTTCCAATACGCAACCTAAAGTAAAATGCCCCACAGCGCTGAGTGCATATAATGCATTCTCTAGTGAAAAACCTTGTTGGCATAAAAAGGCTAATTGATTTTCGAGAGTTTCATACTGTTTTTCTGTAGGCCGTGTACCTAAATGTACTTTTGCTCCATCGCGATGACTTAGTAAAGCACATCTAAAACTTTTAGCGTTATTACGTAAAAAATCTTGCCAGCTTTCCCCTTCTAAAGGGCAAAAGTGAGTATGGTGCCTATCTAACATCTCAATGGCTAAGGCGTCGAGCAAAGCCCGCTTATTTTTTACATGCCAATACAATGTAGGCTGCTCTACACCTAGCTTCTGGGCGAGTTTACGGGTTGTTAAACCTTCGATTCCGACCTCATTAAGCAGCTCTAATGCGCTGTTAATCACTTTACTTTTATCTAATCTAGACATCgTTAATTCCTAATTTTTGTTGACACTCTATCATTGATAGAGTTATTTTACCACTCCCTATCAGTGATAGAGAAAAGTcatatg |

**Supplementary Figures:**

**
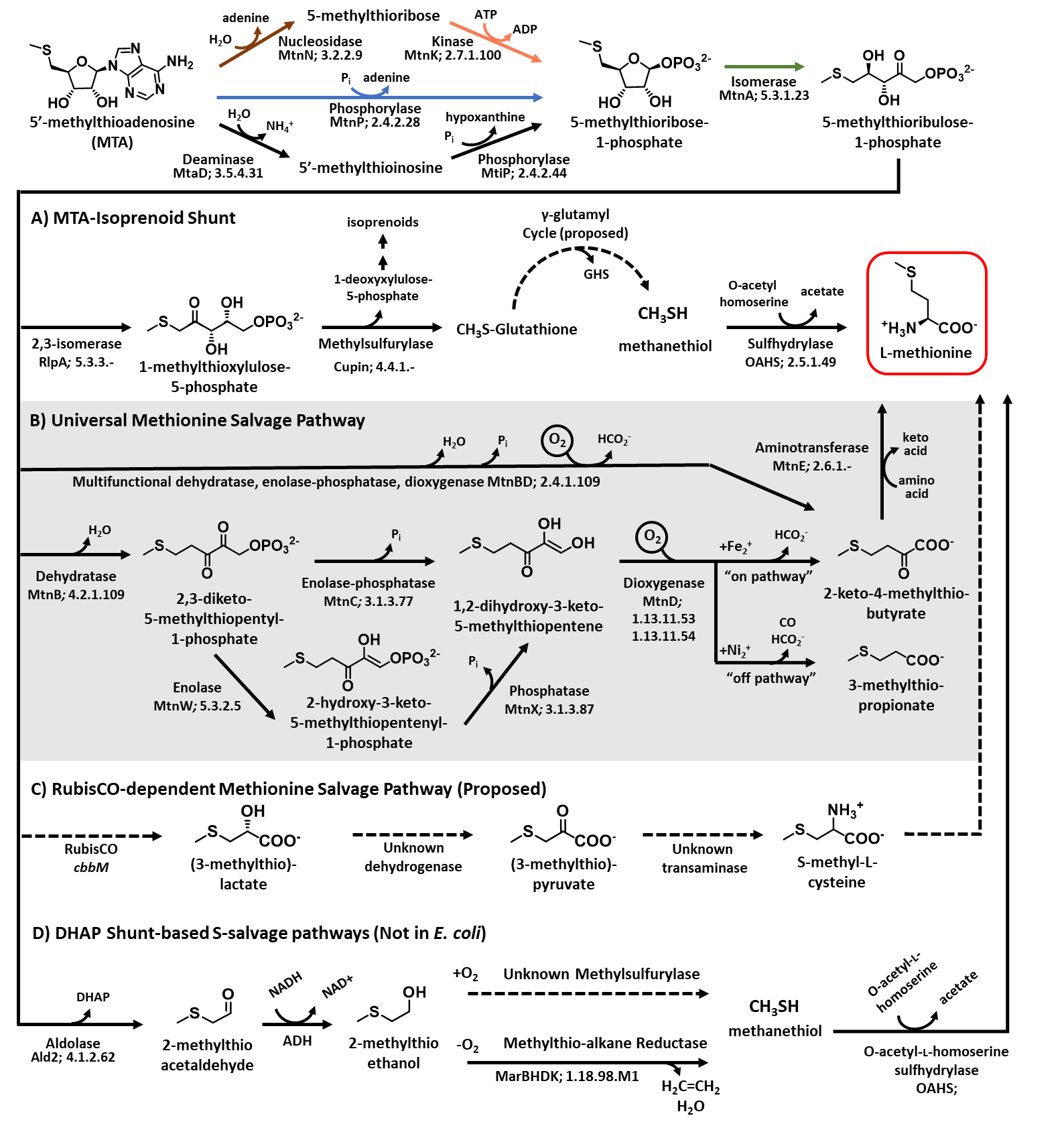
**

**Suppl. Fig. S1. Diversity of sulfur salvage pathways for MTA present in various bacteria.** While some *E. coli* possess the DHAP shunt (MtnN also known as Pfs, MtnK, MtnA, and Ald2) as well as an alcohol dehydrogenase to ultimately convert MTA to 2-methylthioethanol, they are missing gene homologs for other known MTA salvage pathways found in other organisms (A-D). All known sulfur salvage pathways from MTA begin by nucleoside cleavage [1-4]. Some organisms such as *P. aeruginosa* and *M. jannaschii* initially deaminate MTA to enable specific nucleoside cleavage [5-8]. **A)** The MTA-isoprenoid shunt from *R. rubrum* leads to the formation of isoprenoids and methanethiol as the immediate methionine precursor [1, 9-11]. **B)** Microbial variations of the Universal Methionine Salvage Pathway leads to formate and 2-keto-4-methylthiobutyric acid as the immediate methionine precursor [3, 12-14]. **C)** The proposed RubisCO-dependent MTA metabolism pathway from *R. rubrum* [15, 16]. **D)** Several sulfur salvage pathways exist that utilize 2-methylthioacetaldehyde produced by the DHAP shunt. In *R. palustris* under aerobic growth conditions (+O_2_), an unknown methylsulfurylase cleaves 2-methylthioethanol into methanethiol as the immediate methionine precursor [17]. Under anaerobic conditions (-O_2_), organisms with the nitrogenase-like methylthio-alkane reductase reductively cleave 2-methylthioethanol into methanethiol and ethylene [2, 18]. Protein designations and EC numbers are provided below each enzymatic step. ADH, unknown alcohol dehydrogenase.

**Suppl. Fig. S2. pTETTET plasmid map for complementation in *E. coli*.** The dual Tet promoter and *tetR* sequence from the transposon Tn10 [31, 32] was synthesized and modified to contain flanking NdeI and AseI sites. Internal NdeI and AseI sites from TetR were removed by silent substitutions (sequence in Suppl. Table S2). This modified Tet regulon was amplified with primers Pzt1RBS-AseI-F and Pzt1RBS-NdeI-R (Suppl. Table S1) and inserted between the NdeI and AseI sites of pBBRsm2-MCS5 [19] to form plasmid pTETTET. Plasmid map constructed with SnapGene software (from Dotmatics).

**
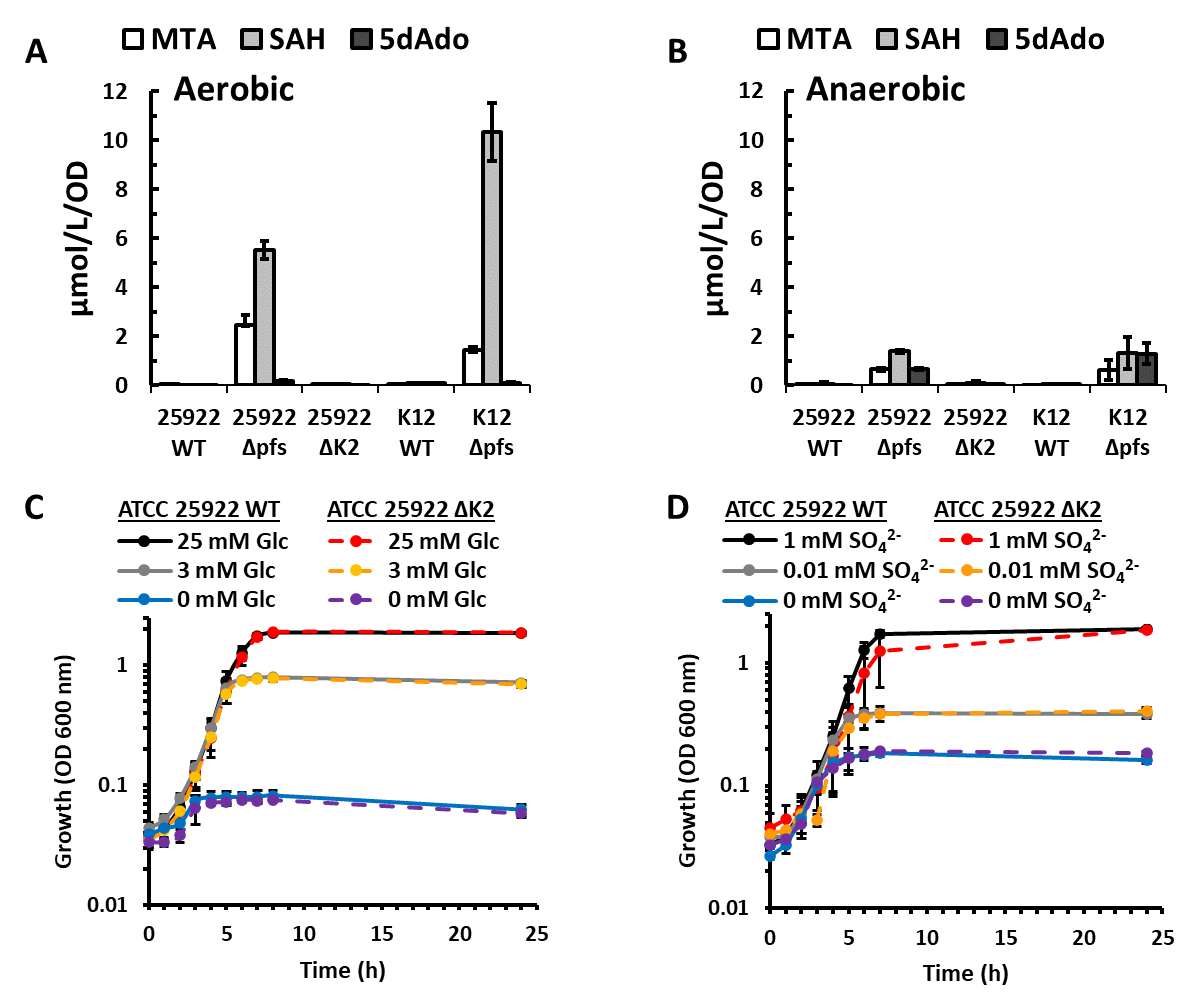
**

**Suppl. Fig. S3. Intracellular SAM byproducts do not need to be salvaged by the DHAP shunt to maintain *E. coli* growth. A-B)** HPLC analysis of internal DHAP shunt metabolites from cultures grown with 25 mM glucose as the sole carbon source with 40 mM sodium nitrate under (A) aerobic or (B) anaerobic conditions. *E. coli* ATCC 25922 or K-12 cultures were harvested at mid-log phase for metabolite analysis. WT, wild type; Δ*pfs*, Pfs nucleosidase deletion strain; ΔK2, *mntK*-*mtnA*-*ald2* deletion strain. **C-D)** Growth of either wild type ATCC 25922 or ATCC 25922 ΔK2 strain under decreasing concentrations of (C) glucose or (D) sulfate. Average and standard deviation error bars are for n=3 independent replicates.


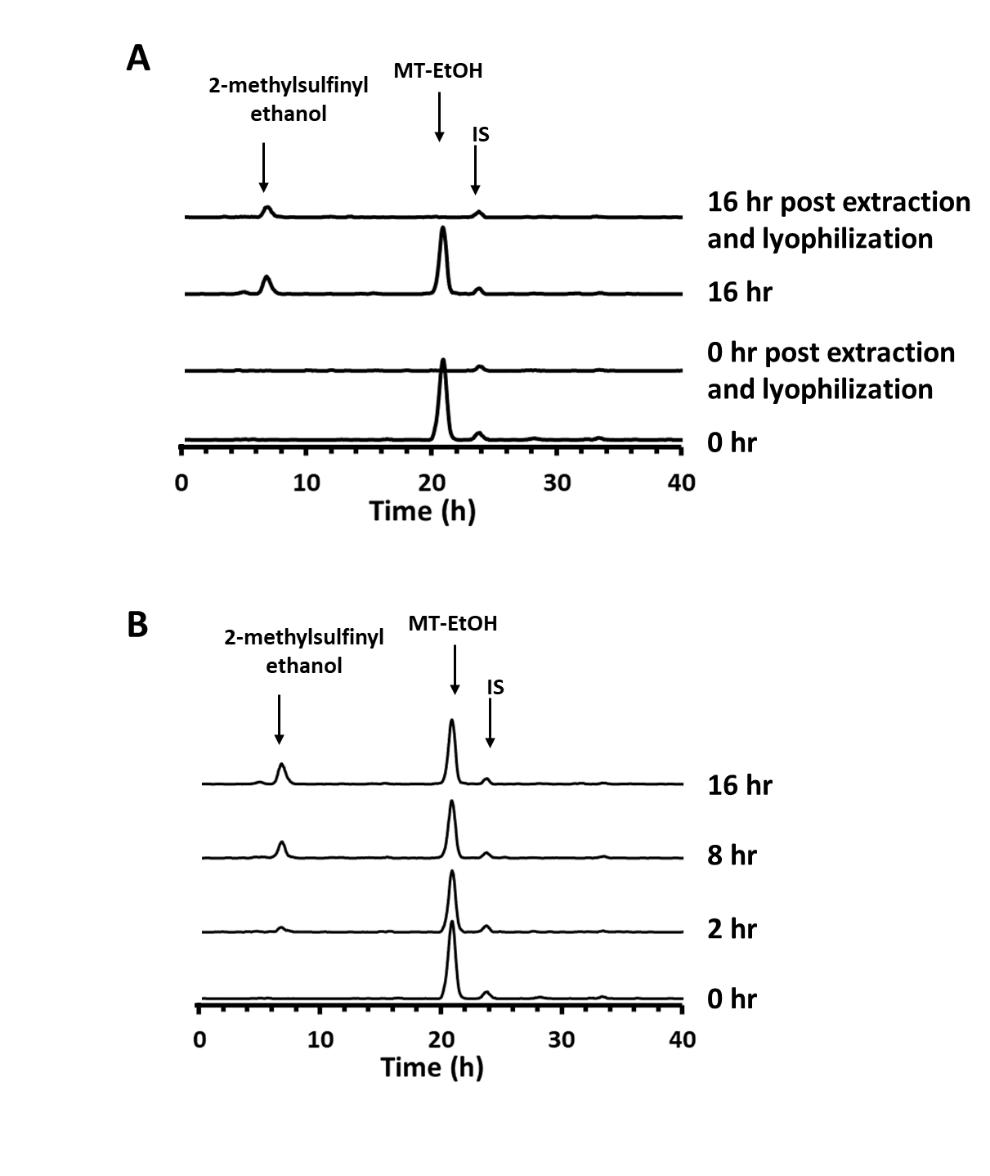


**Suppl. Fig. S4. Reverse Phase HPLC quantification of non-enzymatic oxidation of 2-methylthioethanol to 2-methylsulfinylethanol and selective removal of 2-methylthioethanol by lyophilization. A)** [^14^C-methyl]2-methylthioethanol was synthesized from [^14^C-methyl]-5’-methylthioadenosine as previously described [21] and then incubated in M9 glucose media at 37 °C under aerobic conditions for the indicated amount of time (0 and 16 h) in duplicate. After incubation, one of the two aliquots was combined with an equal volume of acetonitrile as done for extraction of metabolites from cells for LC-MS/MS, centrifuged, and the supernatant lyophilized. Metabolites were resolved by reverse phase HPLC as in Suppl. Fig. S3. **B)** To verify that the oxidation of 2-methylthioethanol to 2-methylsulfinylethanol was temporal, aliquots of [^14^C-methyl]2-methylthioethanol were incubated as in (A) and then resolved by reverse phase HPLC. 2-methylsulfinylethanol, R_T_ = 6.7 min; MT-EtOH – 2-methylthioethanol, R_T_ = 21.7 min; IS - internal standard, R_T_ = 24.0 min.


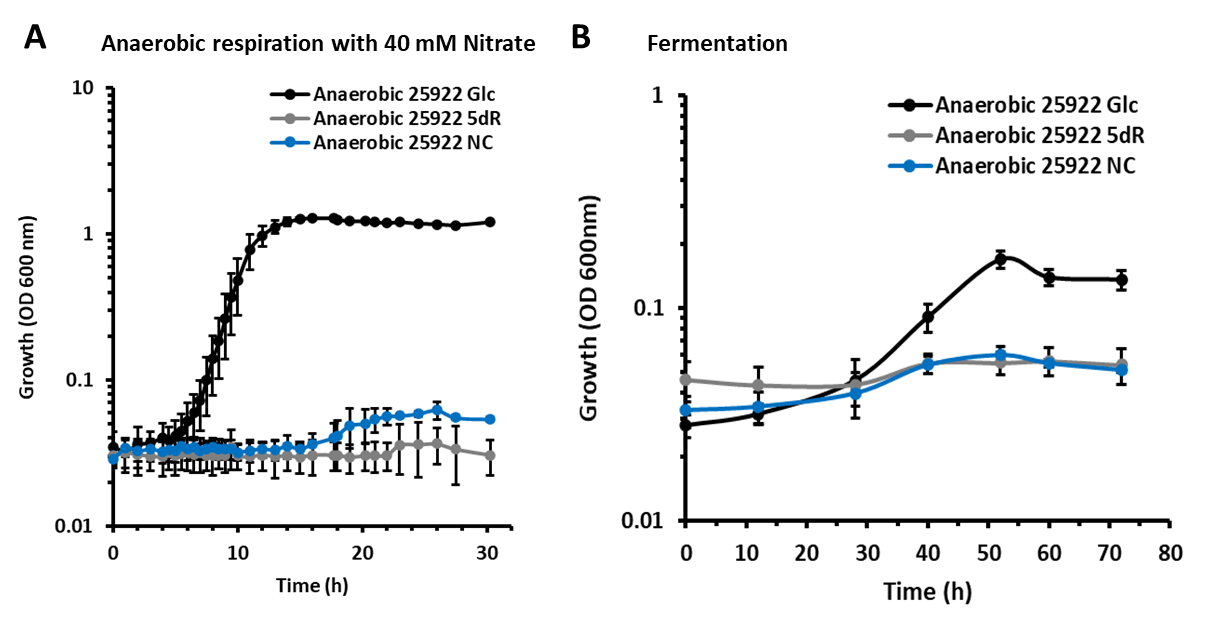


**Suppl. Fig. S5. ATCC 25922 cannot grow anaerobically with 5dR as a sole carbon source via nitrate respiration nor via fermentation.** ATCC 25922 wild type strain was grown anaerobically with (A) 40 mM sodium nitrate or (B) with no terminal electron acceptor and with no carbon (NC), 5 mM glucose (Glc) or 5 mM 5-deoxy-d-ribose (5dR). Starter cultures used for the experiments were grown in 25 mM glucose under the same respective conditions. Averages and standard deviation error bars are for n=3 independent growth experiments.

**
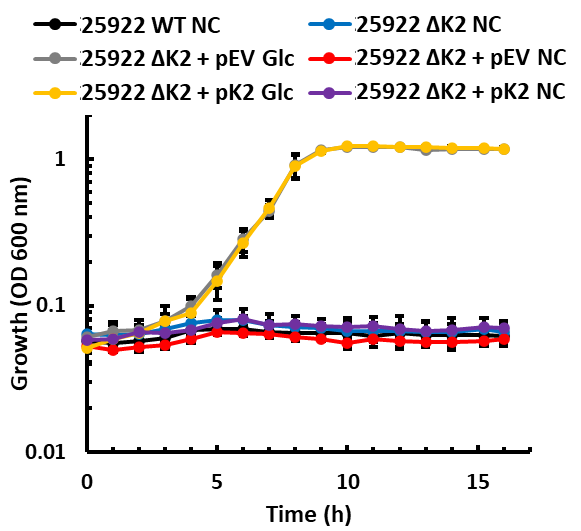
**

**Suppl. Fig. S6. Deletion of DHAP shunt in ExPEC ATCC 25922 does not affect growth on glucose.** ATCC 25922 wild type strain and ATCC 25922 DHAP shunt deletion strain (ΔK2) were grown in no carbon source media (NC) or in the presence of glucose (Glc). Strains were complemented with either the pTETTET empty vector (pEV) or pTETTET expressing the DHAP shunt genes (pK2). Averages and standard deviation error bars are for n=3 independent growth experiments.

**Supplementary Figure References:**

1. Erb, T.J., et al., *A RubisCO-like protein links SAM metabolism with isoprenoid biosynthesis.* Nature chemical biology, 2012. **8**(11): p. 926-932.

2. North, J.A., et al., *Microbial pathway for anaerobic 5′-methylthioadenosine metabolism coupled to ethylene formation.* Proceedings of the National Academy of Sciences, 2017. **114**(48): p. E10455-E10464.

3. Albers, E., *Metabolic characteristics and importance of the universal methionine salvage pathway recycling methionine from 5′‐methylthioadenosine.* IUBMB life, 2009. **61**(12): p. 1132-1142.

4. Parveen, N. and K.A. Cornell, *Methylthioadenosine/S‐adenosylhomocysteine nucleosidase, a critical enzyme for bacterial metabolism.* Molecular microbiology, 2011. **79**(1): p. 7-20.

5. Miller, D., et al., *Identification of a 5′-deoxyadenosine deaminase in Methanocaldococcus jannaschii and its possible role in recycling the radical S-adenosylmethionine enzyme reaction product 5′-deoxyadenosine.* Journal of bacteriology, 2014. **196**(5): p. 1064-1072.

6. Miller, D.V., et al., *Promiscuity of methionine salvage pathway enzymes in Methanocaldococcus jannaschii.* Microbiology, 2018. **164**(7): p. 969-981.

7. Guan, R., et al., *Methylthioinosine phosphorylase from Pseudomonas aeruginosa. Structure and annotation of a novel enzyme in quorum sensing.* Biochemistry, 2011. **50**(7): p. 1247-1254.

8. Guan, R., et al., *Methylthioadenosine deaminase in an alternative quorum sensing pathway in Pseudomonas aeruginosa.* Biochemistry, 2012. **51**(45): p. 9094-9103.

9. Cho, K., et al., *Integration of untargeted metabolomics with transcriptomics reveals active metabolic pathways.* Metabolomics, 2015. **11**: p. 503-517.

10. Warlick, B.P., et al., *1-Methylthio-D-xylulose 5-phosphate methylsulfurylase: A novel route to 1-deoxy-D-xylulose 5-phosphate in Rhodospirillum rubrum.* Biochemistry, 2012. **51**(42): p. 8324-8326.

11. North, J.A., et al., *Metabolic regulation as a consequence of anaerobic 5-methylthioadenosine recycling in Rhodospirillum rubrum.* MBio, 2016. **7**(4): p. e00855-16.

12. Sekowska, A., H. Ashida, and A. Danchin, *Revisiting the methionine salvage pathway and its paralogues.* Microbial biotechnology, 2019. **12**(1): p. 77-97.

13. Sekowska, A., et al., *Bacterial variations on the methionine salvage pathway.* BMC microbiology, 2004. **4**: p. 1-17.

14. Nakano, T., et al., *MtnBD is a multifunctional fusion enzyme in the methionine salvage pathway of Tetrahymena thermophila.* PLoS One, 2013. **8**(7): p. e67385.

15. Dey, S., et al., *In Vivo Studies in Rhodospirillum rubrum Indicate That Ribulose-1, 5-bisphosphate Carboxylase/Oxygenase (Rubisco) Catalyzes Two Obligatorily Required and Physiologically Significant Reactions for Distinct Carbon and Sulfur Metabolic Pathways*♦.* Journal of Biological Chemistry, 2015. **290**(52): p. 30658-30668.

16. Singh, J. and F.R. Tabita, *Roles of RubisCO and the RubisCO-like protein in 5-methylthioadenosine metabolism in the nonsulfur purple bacterium Rhodospirillum rubrum.* Journal of bacteriology, 2010. **192**(5): p. 1324-1331.

17. Miller, A.R., et al., *Two distinct aerobic methionine salvage pathways generate volatile methanethiol in Rhodopseudomonas palustris.* Mbio, 2018. **9**(2): p. e00407-18.

18. North, J.A., et al., *A nitrogenase-like enzyme system catalyzes methionine, ethylene, and methane biogenesis.* Science, 2020. **369**(6507): p. 1094-1098.

19. Schneider, K., et al., *Rhodobacter sphaeroides uses a reductive route via propionyl coenzyme A to assimilate 3-hydroxypropionate.* Journal of bacteriology, 2012. **194**(2): p. 225-232.
